# The diagnostic value and mechanism of miR-127-3p in type 2 diabetes and complications of diabetic nephropathy

**DOI:** 10.1186/s41065-025-00618-x

**Published:** 2025-12-06

**Authors:** Lili Du, Hong Xia, Lingbo Lv, Xin Zhang, Guoxia Luo, Meini Cen

**Affiliations:** 1 Department of Clinical Laboratory, Huangshi Central Hospital, Huangshi, 435000 Hubei China; 2 Huangshi Key Laboratory of Molecular Diagnosis and Treatment of Tumors, Huangshi, 435000 China; 3 Nephrology Department, Shanghai Putuo District People’s Hospital, Shanghai, 200060 China; 4 Department of Endocrinology, Shenzhen Second People’s Hospital, Shenzhen, 518035 Guangdong China; 5 Biology Faculty, Adam Mickiewicz University, Poznań, 61-712 Poland; 6 Department of Medical Laboratory Medicine, Chongqing University Fuling Hospital, No. 2 Gaosuntang Road, Fuling District, Chongqing, 408000 China; 7 Department of Rehabilitation Medicine, The Affiliated Hospital of Youjiang Medical University for Nationalities, No.18, Zhongshan 2nd Road, Youjiang District, Baise, Guangxi, 533000 China; 8 Key Laboratory of Research and Development on Clinical Molecular Diagnosis for High-Incidence Diseases of Baise, No.18, Zhongshan 2nd Road, Youjiang District, Baise, Guangxi, China

**Keywords:** miR-127-3p, ACO2, T2DM, DKD, Oxidative stress, Inflammation

## Abstract

**Background:**

Diabetic kidney disease (DKD) is a serious microvascular complication of type 2 diabetes mellitus (T2DM). miR-127-3p is dysregulated in T2DM, but the specific molecular mechanism remains unclear. We aim to probe the diagnostic value of miR-127-3p and its molecular mechanism in T2DM and DKD.

**Methods:**

This study comprised 218 individuals, including 78 patients with T2DM, 72 patients with DKD and 68 healthy controls. All participants underwent fasting peripheral blood collection. In vitro, we simulated a hyperglycemic environment by treating human mesangial cells (HMC) with high-concentration glucose (HG). Subsequently, RT-qPCR was used to detect the levels of miR-127-3p in serum and HMC. Cell viability and inflammatory cytokine (TNF-α, IL-1β and IL-6) levels were assessed using the CCK-8 assay and ELISA, respectively. The dual-luciferase reporter assay validated the target relationship between miR-127-3p and ACO2.

**Results:**

By comparing baseline clinical characteristics, we identified significant differences among the three groups in high density lipoprotein cholesterol (HDL-C), triglycerides (TG), fasting blood glucose (FBG), glycated hemoglobin A1c (HbA1c), blood urea nitrogen (BUN), estimated glomerular filtration rate (eGFR) and albuminuria. Additionally, miR-127-3p was elevated in T2DM and DKD patients. It could distinguish healthy individuals from T2DM or T2DM from DKD. In HG-induced HMC, miR-127-3p inhibitor elevated the cell viability and the levels of SOD while suppressing the levels of MDA. These effects were abolished by ACO2 silencing. Furthermore, downregulated miR-127-3p reduced the levels of TNF-α, IL-1β and IL-6. sh-ACO2 alleviated the inhibitory effects of miR-127-3p.

**Conclusions:**

Upregulated miR-127-3p was involved in the progression of T2DM and DKD. In HG-induced HMC, down-regulated miR-127-3p improved cell viability and suppressed oxidative stress and inflammatory responses by negatively regulating ACO2.

## Background

Diabetes mellitus (DM) is a metabolic disease that comprises three types: type 1 diabetes mellitus (T1DM), type 2 diabetes mellitus (T2DM) and gestational diabetes mellitus (GDM) [1]. The current population of diabetes patients is huge, which is forecast to grow to 600 million by 2035 and reach 783 million by 2045 [2]. Furthermore, approximately one-third of diabetes patients will go on to develop diabetic kidney disease (DKD) [3]. DKD is a severe microvascular complication of DM and a pivotal factor in morbidity and mortality in T2DM [4]. It is worth noting that DKD is an irreversible chronic pathological change, thus the major goal of therapy is to retard disease progression and control its symptoms. For patients with T2DM, lifestyle changes and metformin remain the first-line treatment options. DKD nursing strategies including but not limited to monitoring blood pressure and blood sugar, educating patients about their disease, ensuring patients eat a low-protein diet, encouraging walking and exercise, and teaching patients medication adherence [5]. However, the precision nursing model (PNM) is more conducive to improving patients’ adherence to treatment and quality of life than traditional nursing models. A study revealed that the implementation of PNM during hemodialysis in patients with DKD effectively improved clinical parameters and reduced the incidence of complications [6]. Moreover, the most important diagnostic and prognostic indicators for DKD are albuminuria and glomerular filtration rate, both of which have significant drawbacks. Therefore, there is a growing need for new biomarkers to predict DKD.

Due to their stability in body fluids, microRNAs (miRNAs) are crucial for diagnosing T2DM and DKD. These miRNAs are associated with DKD pathogenesis by regulating pathways involved in inflammation, fibrosis and metabolic dysfunction [7]. For instance, miR-1281 was up-regulated in T2DM and DKD and can serve as a potential diagnostic marker for T2DM and DKD [8]. miR-1225-3p inhibitor restrained fibrosis and inflammation in renal tissues of DKD mice [9]. Notably, the abnormal expression of miR-127-3p in T2DM attracted our attention [10]. Moreover, miR-127-3p could suppress autophagy to alleviate kidney cell damage in acute kidney injury [11]. And downregulated miR-127-3p facilitated excessive activation of the type I interferon signaling pathway in lupus nephritis [12]. It is evident that miR-127-3p was closely associated with kidney diseases. Nevertheless, the role of miR-127-3p in T2DM and DKD remains unknown.

Our experimental design consists of three main components. (1) Exploring the diagnostic value of miR-127-3p in T2DM and DKD through serum detection. (2) Investigating the effects of miR-127-3p on inflammation and oxidative stress using cell models in vitro. (3) Validating that miR-127-3p regulates these processes via ACO2 through rescue experiments.

## Methods

### Study subjects

The trial has been approved by the ethics committee of Shenzhen Second People’s Hospital. Informed consent has been obtained from the participants.

We recruited 150 patients with diabetes mellitus who visited Shenzhen Second People’s Hospital between October 2023 and February 2025, of whom 78 participants were diagnosed with T2DM and 72 participants were diagnosed with DKD. The control group consisted of 68 healthy individuals. Notably, T2DM patients did not have diabetes complications, including DKD, diabetic retinopathy and diabetic neuropathy. The diagnostic criteria for DKD are T2DM accompanied by persistent albuminuria (≥ 30 mg/24 h) or eGFR < 60 mL/min/1.73 m^2^. Exclusion criteria: (1) Presence of other diabetic complications; (2) Other types of kidney disease; (3) Abnormal liver function and cardiopulmonary function; (4) Severe infection and malignant tumors.

All subjects’ fasting peripheral blood was obtained. After centrifugation at 3000 rpm for 15 min, the serum was kept at -80 °C.

### Cell processing and transfection

Human glomerular mesangial cells (HMC; Procell, China) were grown in DMEM (Thermofisher, USA) containing 10% FBS (Thermofisher, USA) and 1% penicillin-streptomycin (Thermofisher, USA). The control group was treated with 5.5 mM glucose (Thermofisher, USA), and the HG group was treated with 30 mM glucose.

miR-127-3p mimic, miR-17-3p inhibitor, sh-ACO2 and their negative controls were procured from RiboBio (RiboBio, China). The mimic NC and inhibitor NC were negative controls for miR-127-3p mimic and miR-127-3p inhibitor, respectively. sh-NC served as the negative control for sh-ACO2. miR-127-3p mimic and miR-127-3p inhibitor were transfected with Lipofectamine 2000 (Thermofisher, USA), and sh-ACO2 was transfected by Entranster-R4000 (Engreen, China).

### RT-qPCR

Total RNA was isolated with Trizol reagent (Thermofisher, USA). Subsequently, PCR was performed on the Applied Biosystems 7300 Real-Time PCR System (Applied Biosystems, USA) following the protocol of the BeyoFast SYBR Green One-Step qRT-PCR Kit (Beyotime, China). We used U6 as the internal reference gene for miR-127-3p and GAPDH as the internal reference gene for ACO2. The data were calculated via 2^−ΔΔCT^ method.

### Cell viability assay

Cell Counting Kit-8 (Yeasen, China) was employed to quantify cell viability. Cells (5 × 10³ cells/well) were seeded into a 96-well plate and cultured overnight. Following completion of cell culture, fresh medium was added to each well, along with an additional 10 µL of CCK-8 reagent. After 48 h, the OD at 450 nm was tested.

### Assay of oxidative stress levels

Lipid Peroxidation MDA Assay Kit and Total Superoxide Dismutase Assay Kit with WST-8 (Beyotime, China) were utilized to test the content of MDA and SOD.

### ELISA

Inflammatory factor content (TNF-α, IL-1β and IL-6) was tested with an ELISA Kit (Thermofisher, USA).

### Dual-luciferase reporter assay

The binding sites were acquired via ENCORI (https://rnasysu.com/encori/index.php). Subsequently, we inserted the synthesized sequences into the pmirGLO vector (Promega, USA) to construct pmirGLO-ACO2 (ACO2-wt). The plasmid carrying the complementary strand of the binding site was served as a control (ACO2-mut). The aforementioned plasmids were co-transfected into cells with miR-127-3p mimic or miR-127-3p inhibitor. After 48 h, luciferase activity was tested by the Dual Luciferase Reporter Gene Assay Kit (Beyotime, China).

### Statistical analysis

Data were processed in GraphPad Prism 9.3.1 and IBM SPSS Statistics 27 and presented as mean ± standard deviation. The area under the ROC curve was considered as a measure of diagnostic performance. Pearson correlation analysis was employed to identify linear relationships among variables. Three biological replicates were included in each group in the in vitro experiments. Differences between two groups were analyzed using an unpaired t-test. Differences among multiple groups (> 2 groups) were analyzed using one-way ANOVA with Tukey’s post hoc test. *p* < 0.05 was deemed statistically significant.

## Results

### Clinical baseline characteristics

Initially, we evaluated the clinical baseline characteristics of the patients (Table 1). Chi-square tests revealed no notable differences in age and gender (*p* > 0.05) among the healthy, T2DM and DKD groups. One-way ANOVA analysis indicated that there were no differences in body mass index (BMI), systolic blood pressure (SBP), diastolic blood pressure (DBP), total cholesterol (TC), low density lipoprotein cholesterol (LDL-C) and creatinine (*p* > 0.05). Nevertheless, high density lipoprotein cholesterol (HDL-C), triglycerides (TG), fasting blood glucose (FBG), glycated hemoglobin A1c (HbA1c), blood urea nitrogen (BUN), estimated glomerular filtration rate (eGFR) and albuminuria (*p* < 0.001) exhibited significant differences.

**Table 1:** Clinical baseline characteristics of patients

Variables	Control (*n* = 68)	T2DM (*n* = 78)	DKD (*n* = 72)	*P* value
Age (year)	50.41 ± 10.17	51.62 ± 10.24	51.76 ± 11.13	0.71
Gender, male (%)	30 (44.12)	37 (47.44)	34 (47.22)	0.91
BMI (kg/m2)	24.67 ± 3.26	25.12 ± 2.92	24.98 ± 3.40	0.68
SBP (mmHg)	127.14 ± 8.06	127.20 ± 6.94	129.39 ± 4.92	0.08
DBP (mmHg)	76.97 ± 4.86	78.42 ± 6.25	78.74 ± 6.75	0.18
TC (mg/dL)	184.69 ± 8.41	184.51 ± 32.59	188.54 ± 35.24	0.63
LDL-C (mg/dL)	119.00 ± 21.52	116.92 ± 40.76	113.06 ± 31.82	0.55
HDL-C (mg/dL)	61.89 ± 13.72	50.49 ± 15.23	46.90 ± 11.23	< 0.001
TG (mg/dL)	127.09 ± 44.46	208.56 ± 71.83	243.78 ± 91.96	< 0.001
FBG (mmol/L)	5.24 ± 1.30	6.81 ± 0.78	8.39 ± 0.94	< 0.001
HbA1c (%)	5.39 ± 0.94	9.51 ± 2.65	10.75 ± 2.14	< 0.001
BUN (mg/dL)	11.60 ± 2.68	14.92 ± 4.31	20.62 ± 7.98	< 0.001
Creatinine (mg/dL)	0.83 ± 0.16	0.84 ± 0.25	0.89 ± 0.23	0.21
eGFR (mL/min)	110.54 ± 12.60	92.87 ± 20.86	53.96 ± 17.43	< 0.001
Albuminuria (mg/24 h)	2.95 ± 1.19	9.09 ± 3.05	205.98 ± 92.40	< 0.001

*T2DM* type 2 diabetes mellitus, *DKD *diabetic kidney disease patients, *BMI* body mass index, *SBP* systolic blood pressure, *DBP* diastolic blood pressure, *TC* total cholesterol, *TG* triglycerides, *HDL-C* high density lipoprotein cholesterol, *LDL-C* low density lipoprotein cholesterol, *FBG* fasting blood glucose, *HbA1c* glycated hemoglobin A1c, *BUN* blood urea nitrogen, *eGFR* estimated glomerular filtration rate

### The clinical diagnostic value of miR-127-3p

**Fig. 1:**
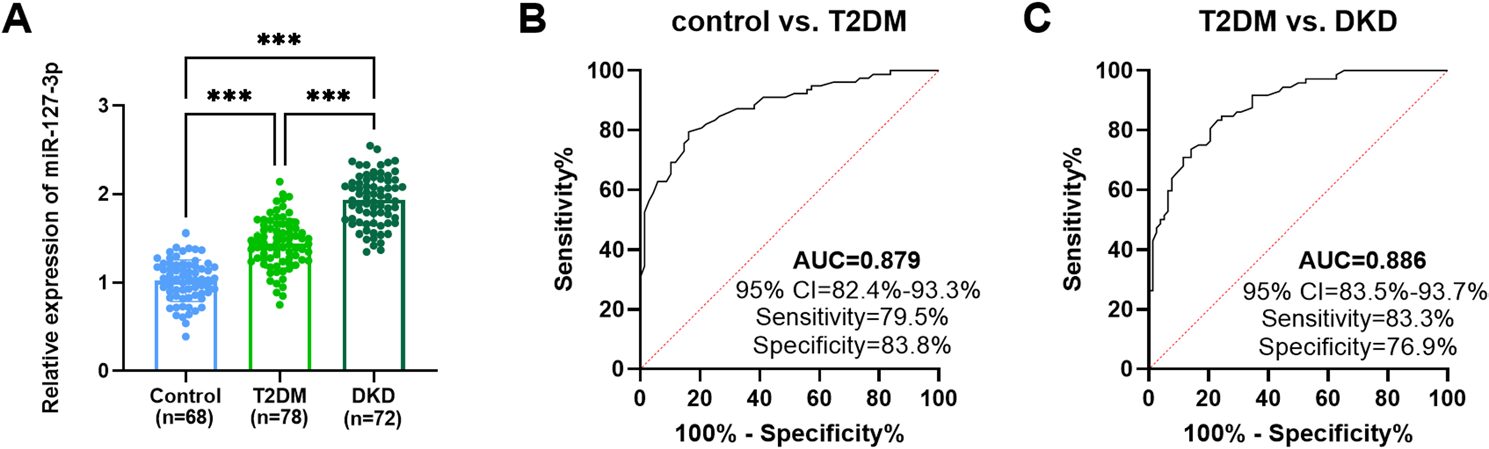
Diagnostic value of miR-127-3p. **A** miR-127-3p content was markedly elevated in T2DM and DKD than in healthy people. ***p < 0.001. **B-C** miR-127-3p could effectively distinguish healthy people from T2DM or T2DM from DKD

Subsequently, we detected the miR-127-3p concentration in the fasting peripheral blood of participants. Compared with the control group, the levels of miR-127-3p were elevated by 0.4-fold in T2DM and by 0.9-fold in DKD (*p* < 0.001; Fig. 1A). Furthermore, ROC curve analysis suggested that miR-127-3p could distinguish healthy individuals from T2DM patients (AUC = 0.879, 95% CI = 82.4%-93.3%, sensitivity = 79.5%, specificity = 83.8%) or T2DM patients from DKD patients (AUC = 0.886, 95% CI = 83.5%-93.7%, sensitivity = 83.3%, specificity = 76.9%; Fig. 1B-C).

### Down-regulated miR-127-3p improved the cell viability and suppressed oxidative stress and inflammation

**Fig. 2:**
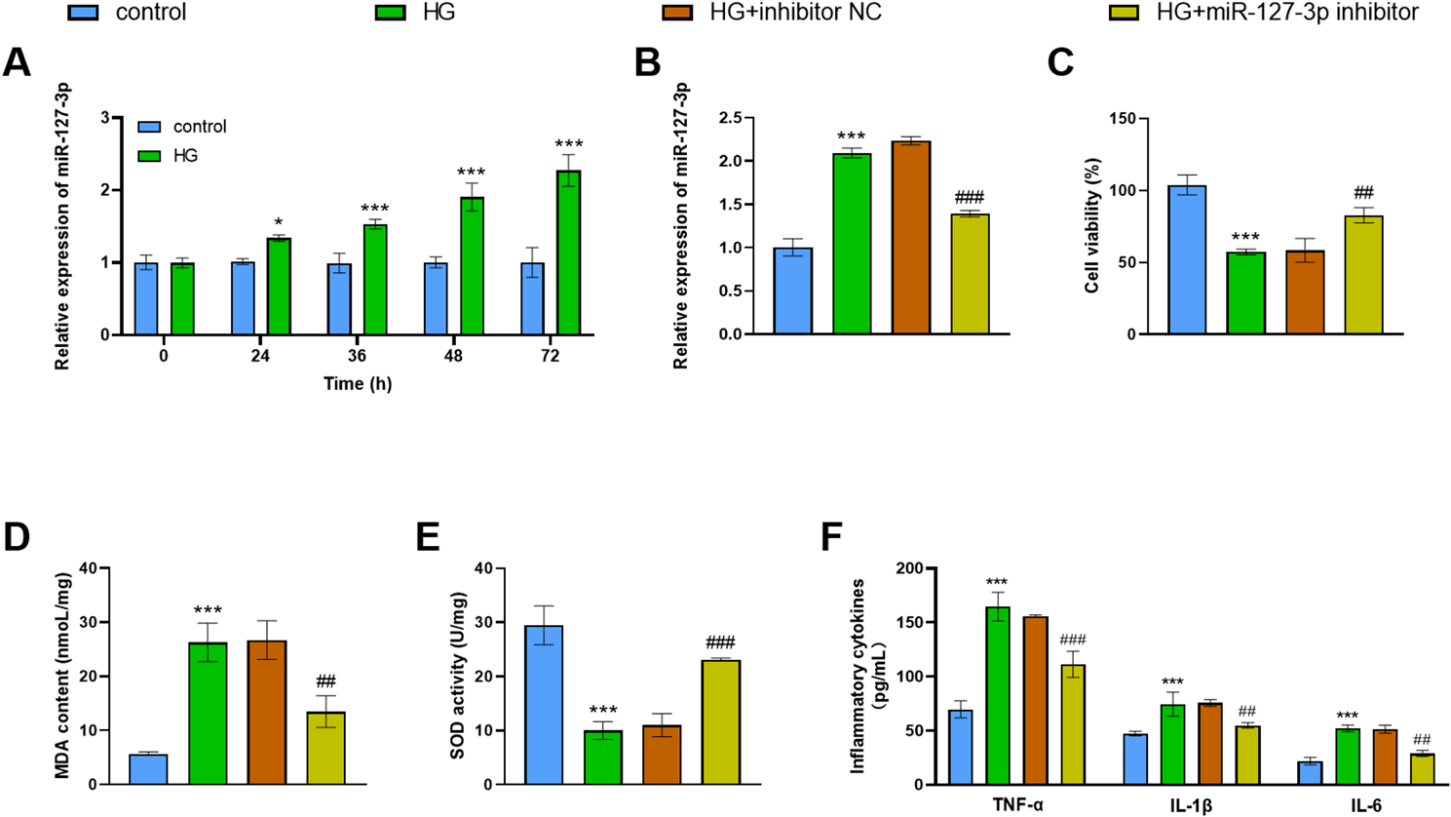
Down-regulated miR-127-3p mitigated HMC damage. **A** miR-127-3p is positively correlated with HG treatment duration. ***p < 0.001, *p < 0.05. **B** HG treatment caused a remarkable elevation in miR-127-3p levels. The miR-127-3p inhibitor reduced miR-127-3p levels in the HG group. ***p < 0.001, vs. control; ^###^*p* < 0.001, vs. HG. **C-F** HG treatment reduced cell viability and SOD levels and elevated MDA and proinflammatory factor levels. This situation could be reversed by suppressing miR-127-3p. ***p < 0.001, vs. control; ^###^*p* < 0.001, ^##^*p* < 0.01, vs. HG

A previous study indicated that hyperproliferation of mesangial cells and extracellular matrix deposition contribute to the progression of DKD [13]. Therefore, we employed glucose-treated HMC to mimic the high-glucose environment of the kidney. Our findings disclosed that the miR-127-3p concentration was proportional to the duration of HG treatment (*p* < 0.05; Fig. 2A). HG exposure elevated the miR-127-3p levels and the miR-127-3p inhibitor dropped the miR-127-3p levels in HG group (*p* < 0.001; Fig. 2B). Moreover, HG treatment reduced cell viability and the content of SOD while upregulating the levels of MDA, TNF-α, IL-1β and IL-6. This condition could be reversed by miR-127-3p inhibitor (*p* < 0.01; Fig. 2C-F).

### miR-127-3p directly targets ACO2

**Fig. 3:**
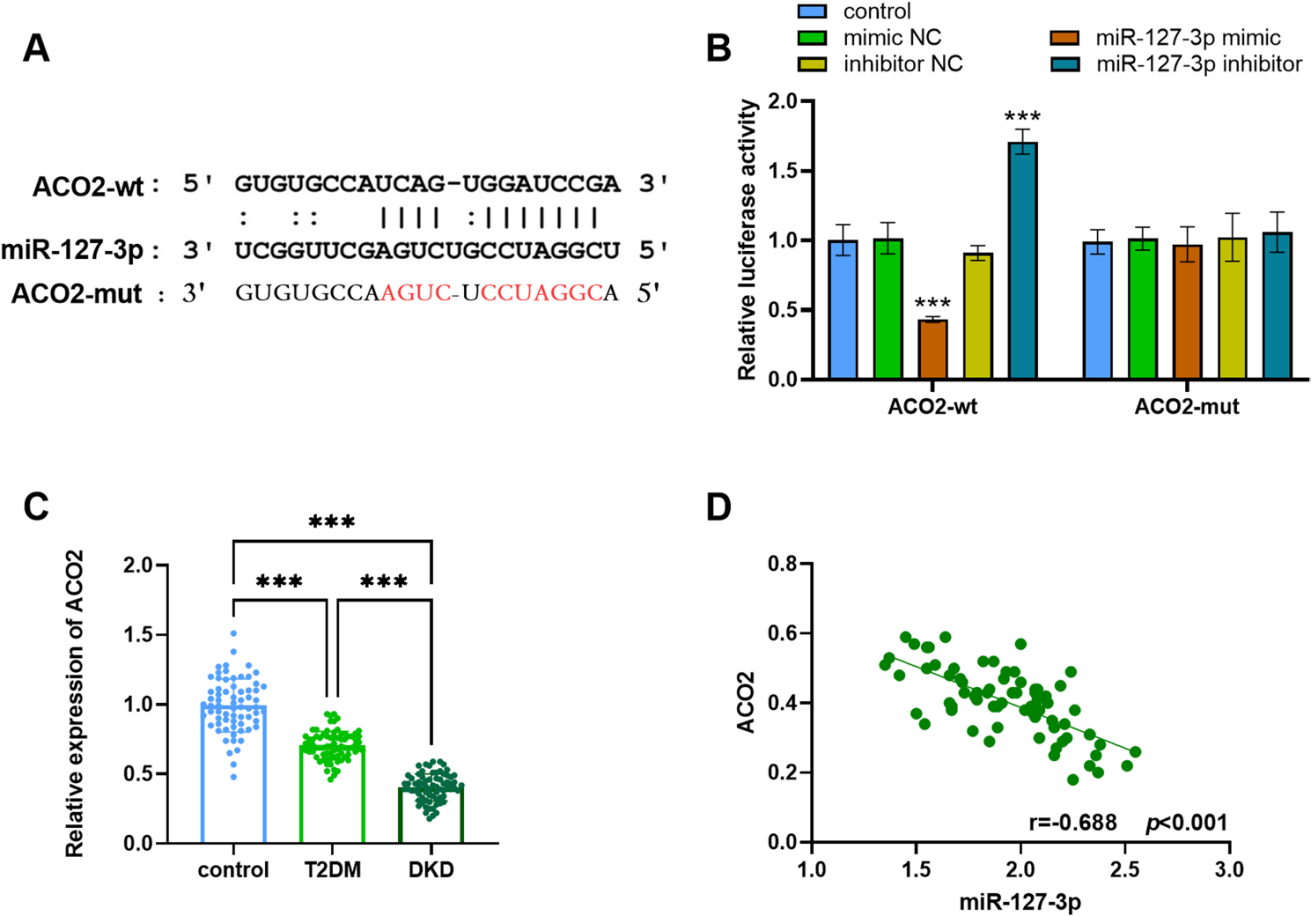
Target relationship between miR-127-3p and ACO2. **A** Binding sites of miR-127-3p and ACO2. **B** In the ACO2-wt group, miR-127-3p mimic repressed luciferase activity. The miR-127-3p inhibitor promoted luciferase activity. No noticeable changes were observed in the ACO2-mut group. ***p < 0.001. **C** miR-127-3p was remarkably diminished in T2DM and DKD patients. ***p < 0.001. **D** The ACO2 concentration was negatively correlated with miR-127-3p

Through the ENCORI database, we identified the binding site between miR-127-3p and ACO2 (Fig. 3A). The luciferase activity was suppressed by miR-127-3p mimic and enhanced by miR-127-3p inhibitor (*p* < 0.001; Fig. 3B). Compared with the control group, the levels of ACO2 were reduced by 0.3-fold in T2DM and by 0.6-fold in DKD (*p* < 0.001; Fig. 3C). And ACO2 content was negatively correlated with miR-127-3p (*r* = -0.688, *p* < 0.001; Fig. 3D).

### Down-regulated miR-127-3p alleviates glomerular damage via negatively regulating ACO2

**Fig. 4:**
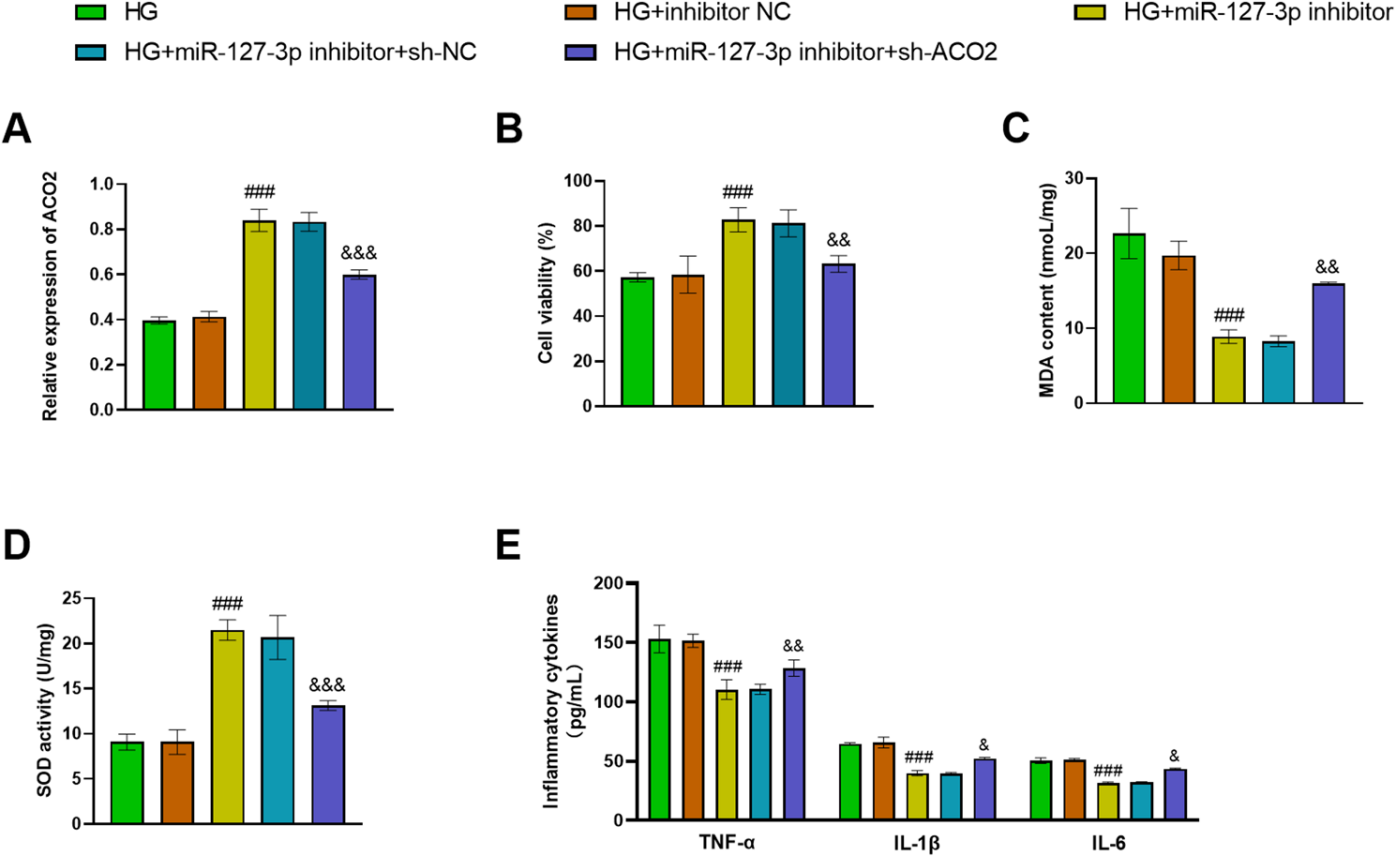
miR-127-3p affects HMC by modulating ACO2. **A** miR-127-3p inhibitor boosted ACO2 content, which was reversed by sh-ACO2. ^###^*p* < 0.001, vs. HG; ^&&&^*p* < 0.001, vs. HG + miR-127-3p inhibitor. **B-E** Down-regulated miR-127-3p improved the survival rate and SOD content and suppressed the MDA and proinflammatory factor content in HG cells. This effect was rescued by sh-ACO2. ^###^*p* < 0.001, vs. HG; ^&&&^*p* < 0.001, ^&&^*p* < 0.01, ^&^*p* < 0.05, vs. HG + miR-127-3p inhibitor

To silence ACO2 expression, we transfected cells with sh-ACO2. The findings uncovered that miR-127-3p inhibitor boosted ACO2 levels in the HG group. sh-AOC2 further reduced the ACAO2 content in the HG + miR-127-3p inhibitor group (*p* < 0.001; Fig. 4A). Moreover, decreased miR-127-3p elevated the cell viability and the levels of SOD, while diminishing the content of MDA and proinflammatory factors in HG cells. This effect could be rescued by inhibiting ACO2 (*p* < 0.05; Fig. 4B-E).

## Discussion

DKD is a manifestation of chronic kidney disease triggered by diabetes, which ultimately leads to ESRD [14]. The specific pathogenesis of DKD is uncertain, but it appears to be caused by a combination of genetic and environmental factors. Such interactions resulted in structural changes in the glomerular capillaries and tubules, including thickening of the glomerular basement membrane, mesangial expansion, thickening of the glomerular basement membrane, disappearance of podocyte foot processes, reduction in podocyte number and density and tubulointerstitial fibrosis [4]. The main goals of DKD therapy are to maintain blood sugar levels, control hypertension, regulate hemodynamics and manage other metabolic disorders [15]. Moreover, proper nursing care could remarkably slow down the progression of disease. Previously, the American Diabetes Association developed a DKD prevention care model aimed at alleviating the onset and progression of DKD with prevention and treatment [16]. Hemodialysis is the primary treatment for DKD. Studies have indicated that receiving humanistic care during hemodialysis treatment effectively minimized negative psychological emotions in patients [17]. And PNM during hemodialysis also improved patients’ clinical indicators and satisfaction [6]. Notably, DKD is primarily characterized by elevated proteinuria and declined glomerular filtration rate (GFR). This is consistent with our findings. By comparison of the clinical baseline characteristics of the enrolled patients, we discovered that serum eGFR content was markedly decreased and albuminuria content was remarkably elevated in DKD patients. Furthermore, HDL-C, TG, FBG, HbA1c and BUN also manifested obvious differences. It demonstrated that the enrolled participants were representative.

The gold standard for diagnosing DKD is renal biopsy. But it is not recommended for patients without proteinuria and/or end-stage renal failure due to the risk of bleeding [18]. Since miRNAs could be obtained from body fluids such as blood or urine, they offer advantages in terms of safety and convenience. Thus, miRNAs have become diagnostic biomarkers for various diseases including DKD [19, 20]. Moreover, previous studies have found that miR-127-3p was up-regulated in atherosclerosis and knee osteoarthritis, which is consistent with our results [21, 22]. In our research, miR-127-3p was markedly up-regulated in T2DM and DKD. It demonstrated good distinguishability between control and T2DM or T2DM and DKD. Hence, we considered that miR-127-3p may be a potential diagnostic marker for T2DM or DKD. To mimic a high-glucose environment in the kidneys, we exposed HMC to HG. It was discovered that miR-127-3p was elevated in HG treated cells, which was consistent with our clinical results. Suppressing miR-127-3p promoted cell viability and suppressed oxidative stress and inflammatory responses in the HG group. This was similar to previous experimental results obtained in myoblasts and macrophages [23, 24].

Aconitases perform a pivotal role in the interaction among citrate and iron metabolism [25]. Two subtypes of aconitase existed in mammalian cells: mitochondrial aconitase (ACO2) and bifunctional cytosolic enzyme (ACO1) [26]. Studies have indicated that ACO2 expression was reduced in renal cell carcinoma and acute kidney injury [27, 28]. And low levels of ACO2 were detected in diabetic urine exosomes [29]. Thus, we speculated that ACO2 was strongly associated with the progression of T2DM and DKD. Moreover, a study has reported that ACO2 knockdown elevated IL-6 and IL-8 levels in HELA cells [30]. In a Parkinson’s mouse model, overexpression of ACO2 mitigated α-synuclein-induced mitochondrial dysfunction and cytotoxicity [31]. In this article, we measured a low ACO2 level in the serum of T2DM and DKD patients, which is consistent with the findings of previous studies. Down-regulated ACO2 could mitigate the elevated cell viability and the repression of oxidative stress and inflammation evoked by miR-127-3p inhibitor. Therefore, we speculate that miR-127-3p may affect cell viability, oxidative stress and inflammation via ACO2, thereby aggravating T2DM and DKD.

This study has several limitations that should be considered. First, the majority of our patients originated from the vicinity of the hospital and had limited numbers, which undermined the reliability of the results. Future studies will continue to expand both the sample size and the sources of samples. Second, the mechanism by which miR-127-3p regulated ACO2 was only validated in cell lines. In vivo experiments were lacking. Future work will involve further validation in animal models or human subjects. Third, this study established that miR-127-3p was a key upstream regulator of ACO2. However, the complexity of gene regulatory networks suggests that ACO2 may serve as a central target within a post-transcriptional regulatory network involving multiple miRNAs. Therefore, systematically identifying other miRNAs regulating ACO2 and deeply analyzing its downstream signaling pathways will be key future research directions for our team.

## Conclusions

In summary, our findings confirmed that upregulated miR-127-3p was involved in the progression of T2DM and DKD. In HG-induced HMC, down-regulated miR-127-3p enhanced cell viability and inhibited oxidative stress and inflammatory responses by negatively modulating ACO2. It provides alternative diagnostic biomarkers for T2DM and DKD and potential therapeutic targets for their future treatment.

## Data Availability

The datasets used and/or analysed during the current study are available from the corresponding author on reasonable request.

## Abbreviations

DKD: Diabetic kidney disease
T2DM: Type 2 diabetes mellitus
HG: High-concentration glucose
DM: Diabetes mellitus
GDM: Gestational diabetes
PNM: Precision nursing model
